# Biological Weapons Defense: Infectious Diseases and Counterbioterrorism

**DOI:** 10.3201/eid1204.060090

**Published:** 2006-04

**Authors:** Daniel R. Lucey

**Affiliations:** * Georgetown University School of Medicine, Washington, DC, USA

**Keywords:** AIDS, Asia, HIV

This insightful text, complete with an eBook version on CD-ROM, is edited by 3 scientists from the Department of Defense (DOD) and includes 58 contributors and forewords by David Franz and Mathew Meselson. The 25 chapters are divided into 4 sections: 1) Preparation and Military Support for a Possible Bioterrorism Incident, 2) Medical Countermeasures and Decontamination, 3) Emerging Threats and Future Preparation, and 4) Diagnostic Development for Biowarfare Agents.

Threats discussed include plague, glanders, Q fever, filoviruses (as a specific example of "a world aswarm with viral zoonoses"), anthrax, smallpox, brucellosis, botulism, and ricin. Information is also provided on genetically-engineered protein toxins, as well as genetic fingerprinting for forensic studies and the use of genomics for the agents of tularemia, brucellosis, and clostridial gas gangrene.

Notably, several chapters are devoted to critical topics that are often not found in other books. For example, 2 chapters on aerosol pathogenesis and "Biological Weapons Defense: Effect Levels" are particularly relevant given the US Cities Readiness Initiative that involves planning for an aerosol attack with anthrax or another agent in US metropolitan areas. Similarly valuable is the chapter on decontamination because it provides insight on how to respond to the challenge of recreating a safe environment in which to live and work after a biological attack.

The 25-page chapter on the Global Emerging Infections System (GEIS) of the DOD as it applies to biodefense is well written. Surveillance systems used by DOD-GEIS are described, from the Electronic Surveillance System for the Early Notification of Community-based Epidemics to newer systems, along with ways to integrate DOD and civilian surveillance systems. Other particularly useful chapters that encompass multiple present and potential future biological threats include those on diagnostics. These 3 chapters focus on explaining biological threat identification systems, DNA-based pathogen identification, and immune response–based assays. Taken together, the 25 chapters of this book are a welcome addition to the growing field of counterbioterrorism and complement well the mostly clinical publications already in print.

